# Designing a Tablet-Based Software App for Mapping Bodily Symptoms: Usability Evaluation and Reproducibility Analysis

**DOI:** 10.2196/mhealth.8409

**Published:** 2018-05-30

**Authors:** Till-Ansgar Neubert, Martin Dusch, Matthias Karst, Florian Beissner

**Affiliations:** ^1^ Somatosensory and Autonomic Therapy Research Institute for Diagnostic and Interventional Neuroradiology Hannover Medical School Hannover Germany; ^2^ Section Pain Medicine Clinic of Anaesthesiology and Intensive Care Medicine Hannover Medical School Hannover Germany

**Keywords:** pain drawing, symptom drawing, body outline, usability testing, reproducibility, tablet computers, eHealth, app, chronic pain

## Abstract

**Background:**

Symptom drawings are widely used as a qualitative and quantitative method of assessing pain symptoms for both clinical and research purposes. As electronic drawings offer many advantages over classical pen-and-paper drawings, the last years have seen a shift toward tablet-based acquisition of symptom drawings. However, software that is used in clinical care requires special attention to usability aspects and design to provide easy access for physically impaired or elderly patients.

**Objective:**

The aims of this project were to develop a new tablet-based software app specifically designed to collect patients’ and doctors’ drawings of pain and related bodily symptoms and test it for usability in 2 samples of chronic pain patients (Aim 1) and their treating doctors (Aim 2) as well as for test-retest reliability (Aim 3).

**Methods:**

In 2 separate studies, symptom drawings from 103 chronic pain patients and their treating doctors were collected using 2 different versions of the app. Both patients and doctors evaluated usability aspects of the app through questionnaires. Results from study 1 were used to improve certain features of the app, which were then evaluated in study 2. Furthermore, a subgroup of 25 patients in study 2 created 2 consecutive symptom drawings for test-retest reproducibility analysis. Usability of both app versions was compared, and reproducibility was calculated for symptom extent, number of symptom clusters, and the whole symptom pattern.

**Results:**

The changes we made to the app and the body outline led to significant improvements in patients’ usability evaluation regarding the identification with the body outline (*P*=.007) and the evaluation of symptom depth (*P*=.02), and the overall difficultness of the drawing process (*P*=.003) improved significantly. Doctors’ usability evaluation of the final app showed good usability with 75.63 (SD 19.51) points on the System Usability Scale, Attrakdiff 2 scores from 0.93 to 1.41, and ISONORM 9241/10 scores from −0.05 to 1.80. Test-retest analysis showed excellent reproducibility for pain extent (intraclass correlation coefficient, ICC=0.92) and good results for the number of symptom clusters (ICC=0.70) and a mean overlap of 0.47 (Jaccard index).

**Conclusions:**

We developed a tablet-based symptom drawing app and improved it based on usability assessment in a sample of chronic pain patients and their treating doctors. Increases in usability of the improved app comprised identification with the body outline, symptom depth evaluation, and difficultness of the drawing process. Test-retest reliability of symptom drawings by chronic pain patients showed fair to excellent reproducibility. Patients’ usability evaluation is an important factor that should not be neglected when designing apps for mobile or eHealth apps.

## Introduction

Bodily symptoms, such as pain, headache, discomfort, or paresthesias, are among the most common reasons to see a doctor [1,2]. Quantification of these symptoms has been challenging ever since because of their purely subjective nature, often leaving patient self-report as the only available source of information. Common tools to measure bodily symptoms include questionnaires [3], rating scales [4], and symptom drawings (better known as pain drawings [5] or discomfort drawings). In the latter, the patient receives an outline of the human body or parts thereof and marks or shades the location and distribution of his different symptoms. Such drawings can then be used to extract features such as the body area affected by the symptom, the number of sites, or the average intensity. The spatial distribution of symptoms may also carry valuable information for diagnosis, such as patterns of segmental or peripheral innervation or association with the location of internal organs.

Several groups have developed symptom drawing approaches that were based on tablet computers [6-11]. Such electronic drawings have many advantages over pen-on-paper drawings, the most important being the ability to analyze drawings right after their completion without the need for prior digitization. Of particular interest are tablets with an electronic pen (stylus) as they have 2 main advantages: a much higher precision than drawing with the finger [12,13] and high similarity with pen-on-paper drawings [7]. High reproducibility of electronic drawings has been validated by Barbero et al for chronic low back and neck pain and acute induced pain [6,14].

The aims of this project were to develop a new tablet-based software app and test it for usability in 2 samples of chronic pain patients (Aim 1) and their treating doctors (Aim 2) as well as for test-retest reliability (Aim 3). This app is specifically designed to collect patients’ and doctors’ drawings of pain and related bodily symptoms. Aiming toward high drawing precision, the app contains 4 different views of the human body, and the drawings were collected on a stylus-based tablet.

Therefore, we developed and tested a prototype app (study 1) following in part the suggested design guidelines by Jaatun et al, that is, using action buttons instead of icons, limiting written textual instruction, avoiding rapid changes on the screen, and using a paper metaphor [11]. Usability results, obtained through questionnaires and user observation, led to several improvements of the user interface and other parts of the app, which were then tested again in a similar sample (study 2). Using the improved version of the app, we further conducted a test-retest analysis of symptom drawing reproducibility in chronic pain patients (pain duration ≥3 months).

## Methods

### Study Design

The project comprised 2 consecutive studies: study 1 aimed at evaluating the usability of a prototype of our app. Study 2 was designed to evaluate the final app with all improvements that had been made. The final app version was also used to assess reproducibility of our symptom drawing approach in a test-retest design. The reproducibility study is reported according to the guidelines for reporting reliability and agreement studies (see Multimedia Appendix 1) [15].

Participants of both studies were chronic pain patients and their treating doctors from a pain outpatient department. The project was conducted in accordance with the Declaration of Helsinki and had been approved by the Ethical committee of Hannover Medical School (#2987-2015). All participants were informed about the purpose of the project and gave written informed consent.

Patients were asked to draw their pain and related symptoms before their appointment with the doctor. All of them were using the app for the first time. Following data entry, each patient filled out a usability questionnaire and continued with the routines of the pain outpatient department, namely, filling out standard pain questionnaires and having the appointment with the clinician.

Doctors were asked to enter the findings of their anamnesis and bodily examination during or shortly after seeing the patient. All participating doctors had been briefly trained by one of the authors (TN) on how to use the app.

### Study Participants

#### Pain Patients

In both studies, participants were recruited consecutively from patients of the Pain Outpatient Department of Hannover Medical School. Inclusion criteria were chronic pain (pain duration of ≥3 months), age ≥18 years (legal age in Germany), physical ability to draw symptom drawings on a tablet personal computer (PC), and ability to give written informed consent. Due to our consecutive recruiting approach, our sample should reflect the normal composition of patients in outpatient departments similar to ours.

We screened 70 patients in study 1, of which 52 were included, 15 declined participation, and 3 had to be excluded because they did not fulfill the inclusion criteria. In study 2, we screened 58 patients, of which 51 were included, 5 declined participation, and 2 were excluded because they did not fulfill inclusion criteria.

The mean age was 56.2 (SD 16.1) years in study 1 and 60.4 (SD 15.7) years in study 2. There were no significant differences between both study populations regarding age, sex, body mass index, educational level, number of pain clusters, years of pain treatment, number of previous therapeutic consultations, and usage frequency of tablet computers or comparable devices (Table 1).

**Table 1:** Demographics of our study populations.

Characteristic		Study 1	Study 2	*P* value^a^
Age (years), mean (SD)		56.2 (16.1)	60.4 (15.7)	.19
**Age range, n (%)**				
	18-39	9 (17)	4 (8)	
	40-59	21 (40)	24 (47)	
	60-79	20 (38)	16 (31)	
	80+	2 (4)	7 (14)	
Women, n (%)		32 (62)	34 (67)	.59
BMI^b^ (kg/m²), mean (SD)		27.6 (7.4)	27.0 (6.8)	.67
Education level ISCED^c^ 1997, mean (SD)		2.7 (1.1)	2.4 (0.8)	.25
**Number of pain clusters, mean (SD)**				
	Front	3.7 (4.5)	5.5 (7.3)	.12
	Back	3.6 (3.4)	4.9 (5.7)	.16
	Left	2.9 (3.4)	4.5 (5.2)	.06
	Right	2.7 (3.0)	4.2 (5.4)	.07
Years of pain treatment, mean (SD)		4.0 (1.8)	3.8 (2.2)	.70
Number of previous therapeutic consultations, mean (SD)		10.2 (11.1)	14.1 (17.7)	.18
**Usage of comparable electronic devices, n (%)**				**.18**
	Daily	30 (58)	32 (64)	
	3-4 times/week	6 (12)	6 (12)	
	1-2 times/week	4 (8)	2 (4)	
	1-2 times/month	0 (0)	0 (0)	
	Almost never	7 (13)	1 (2)	
	Never	5 (10)	9 (18)	

^a^Two-tailed *t* test or chi-square test.

^b^BMI: body mass index.

^c^ISCED: International Standard Classification of Education.

#### Doctors

All doctors who evaluated the app were anesthesiologists with at least 5 years of clinical experience. Moreover, 6 of them participated in study 1, of which 2 were pain specialists and 4 were in training for pain specialization. The specialists examined 48 of the study patients, whereas those in training examined 30.

Of the 4 anesthesiologists involved in study 2, 2 were pain specialists and 2 were in training for pain specialization. This time the specialists examined all 51 patients, of which 35 were additionally examined by a specialist in training.

### Tablet Computer

All symptom drawing data were collected on a Samsung Galaxy Note 2014 edition 10.1 (SM-P600) tablet computer running on Android 4.1.2 (study 1) or Android 5.1.1 (study 2). This tablet has a 10.1-inch touch screen with a resolution of 800×1280 pixels and is equipped with an electronic pen (stylus) that was used for all data entry. In contrast to entering data by finger, which uses the capacitive touchscreen, the tablet records stylus interactions with a separate inductive digitizer, which allows for a higher resolution while eliminating unwanted activation of the screen, for example, by the palm.

### Software App

#### General Design

**Figure 1:**
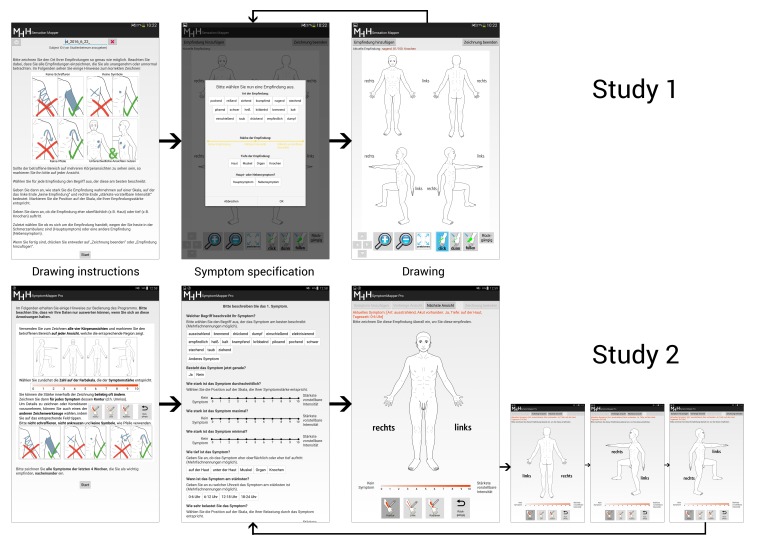
General structure of both app versions. Users were first instructed on how to make a correct symptom drawing (drawing instructions). Then, an iterative process was started, in which users characterized each of their symptoms (symptom specification). Finally, users were asked to mark the location and extent of the symptom on a body outline (drawing).

The software app was organized into 3 different modules: drawing instructions, symptom specification, and drawing (Figure 1). App versions for patients and doctors used the same modules but with different content. In the following sections, we will describe all elements that were left unchanged between studies 1 and 2.

##### Drawing Instructions

The first screen instructed the user how to make a correct symptom drawing. Following the suggested design guidelines by Jaatun et al [11], we used a paper metaphor and limited the written textual instructions as much as possible. Central elements were instructions to draw every symptom or finding that users found disturbing or abnormal and to draw it on each view of the body outline showing the respective body region. Users were further asked to color every point of the body outline where a certain symptom or finding was present. Other possible ways to mark a body region, such as hatching, ticking, or marking by symbols, like arrows, were explicitly prohibited. Finally, users were told to specify the symptom or finding by choosing descriptors from a list. Then, the use of the visual analog scale (VAS) and the rating of depth was explained.

##### Specification of Symptoms

After acknowledging the drawing instructions, a new screen was displayed asking users to specify any pain-related symptom in an iterative process. They were first asked to choose the type of sensation from the following list of descriptors (in German): burning, cold, cramping, dull, electric, heavy, hot, numb, pressing, pricking, radiating, shooting, stabbing, tearing, tender, throbbing, tingling, and tugging. In the next step, they rated the intensity of the sensation on a VAS, ranging from “no sensation” to “strongest imaginable intensity.” Next, they entered the perceived depth of the sensations by choosing one of the following descriptors: skin, muscle, organ, and bone. The initial version of the app contained an additional classification of the symptom into major or minor symptom. Following this textual specification, the drawing module was initialized.

##### Specification of Diagnostic Findings

The doctors’ version of the app had an additional variation of the symptom specification module to enter common diagnostic findings in a bodily examination. This screen contained German translations of the following findings: allodynia, anesthesia, atrophy, dysesthesia, hyperalgesia, hyperhydrosis, and hypesthesia. The VAS and depth rating was similar to that of the symptom specification module.

##### Drawing Module

In the final module, users were shown a body outline to draw the location of the symptom or finding specified in the previous module. It had been specifically developed for this purpose based on photographs of a human body. The sex of the initial version was undetermined. Although previous screens allowed data entry by finger, drawings could only be made using the tablet’s stylus. Users were free to choose from a set of drawing tools (line, autofilled shape, or undo). Drawing was restricted to within the borders of the body outline. After finishing the drawing, users could either choose to end data entry or to add another symptom, which would bring them back to symptom specification.

#### Improvements

We made several improvements to the graphical user interface (GUI) of the prototype app, most of which were inspired by the results of the usability assessment of study 1 as well as requests from the doctors. Furthermore, we completely abandoned the use of pop-up windows as suggested by Jaatun et al [11].

##### Drawing Instructions

Changes to the drawing instructions largely reflect the changes made to the other modules, for example, the newly added VAS of the drawing module (see below). In addition, we added a short explanation of the different drawing tools. As a general rule, we tried to move as much information as possible from instructions to symptom specification, because it seemed a more natural place to explain the choice of descriptors, symptom depth, and the use of the VAS and reduced the amount of information that needed to be memorized by the user. Finally, we reduced ambiguity of the app results for patients by specifying the time interval of the symptoms to be drawn (last 4 weeks).

##### Specification of Symptoms

Many patients asked for the possibility to choose more than one descriptor for the single symptom, which is why we added this feature in the final version. Patients were also allowed to add their own descriptors if they were not happy with the available choices. Furthermore, the list of depth descriptors was changed following patients’ requests. As many of them found it difficult to localize the depth of their symptoms in either “skin” or “muscle,” the term “skin” was split into “on the skin” and “beneath the skin,” whereas the other terms were left unchanged.

Symptom specification was expanded relative to study 1, which was largely motivated by considerations other than usability. Briefly, we asked for each symptom for the maximal and minimal intensity, if the symptom was currently present, the time of day when the symptom was worst (in intervals of 6 hours), and the perceived burden associated with the symptom. The perceived burden replaced the classification into major or minor symptom, because for the majority of the patients all symptoms were rated as major symptom. Maximal and minimal symptom intensity as well as perceived burden were rated on a VAS ranging from 0 (“no symptom” and “no burden”) to 10 (“strongest imaginable intensity” and “strongest imaginable burden”) and anchored by numbers from 0 to 10. All changes were applied after consulting the participating doctors.

##### Specification of Diagnostic Findings

In study 2, finding specification for doctors was also expanded. Besides the possibility to choose multiple symptom descriptors and depth categories, the second app contained an expanded list of findings (Table 2). In addition, doctors were able to add their own descriptors, and the depth descriptor were modified in the same way as for the patients’ version.

##### Drawing Module

Several profound design changes were made to the drawing module, all inspired by user feedback. To allow users to indicate local differences in symptom or finding intensity, we added a VAS to the drawing module. Different intensities in the drawing were indicated by different saturation values of the drawing color. We also changed the body outline and the way it was presented. First, we added the possibility to choose the gender of the outline (female, male, not specified). Sex-related changes to the outline were kept as small as possible to maintain comparability between the drawings from the different sexes.

Second, we changed the mode of presentation. While the four views (front, back, left, right) of the body outline had been presented on one screen in the prototype (Figure 1), the final app showed each view on a separate consecutive screen and users had to click through each of them. The motivation for this was that it allowed us to double the available screen area for drawing and to encourage patients to make use of all available body views.

We also changed the controls (ie, icons) at the bottom of the screen. Buttons for zoom (magnification glass) and scrolling (arrows) were removed because they had only been used by a minority of users. Furthermore, we reduced the line drawing tool to one thickness and added an eraser tool.

### Outcome Measures

#### Usability Assessment

Usability of the app and data acquisition method were assessed separately in patients (Aim 1) and doctors (Aim 2).

**Table 2:** Specification of diagnostic findings for doctors (used in study 2).

Category	Diagnostic finding
Pain/paresthesia	Burning, cold, cramping, dull, electric, heavy, hot, numb, pressing, pricking, radiating, shooting, stabbing, tender, throbbing, tingling, tugging, other
Symptom depth	On the skin, beneath the skin, muscle, organ, bone
Skin (sensitivity)	Allodynia, analgesia, anesthesia, dysesthesia, hypoesthesia, hyperalgesia, hypoalgesia, pallanesthesia, pallhypesthesia, thermanesthesia, thermhypesthesia, other
Skin (perfusion)	Cyanosis, hyperthermia, hypothermia, pallor, redness, swelling, other
Skin (autonomic)	Anhidrosis, atrophy, hyperhidrosis, hypertrophy, piloerection, other
Muscle	Allodynia, atrophy, disturbed proprioception, fasciculation, hyperalgesia, hypotonia, muscular defense, myogelosis, rebound tenderness, rigor, spasm, tenderness, other
Organ	Tenderness, hypertrophy, induration, other

##### Patients’ Evaluation

To evaluate the app and the tablet-based data acquisition, we designed a usability questionnaire. It contained a common part aimed at comparing usability between studies 1 and 2 and an individual part with items specific to each of the studies. The common part consisted of 8 Likert-type questions (possible answers from 0 to 10) and 2 open questions with free text answers. The individual part contained 3 dichotomous and 1 multiple-choice question for study 1 and one Likert-type question for study 2. A translated version of the 2 questionnaires can be found in Multimedia Appendix 2.

##### Doctors’ Evaluation

We observed doctors’ evaluation during data entry at several timepoints of the project and asked them to report any problems or ideas for improvement. These were collected in a list and later analyzed. Furthermore, a meeting with the participating doctors was arranged after the first study to discuss the study results and plans for app improvements.

At the end of study 2, we evaluated usability from the doctors’ perspective through a Web-based survey comprising the following questionnaires:

The System Usability Scale (SUS) [16] is a questionnaire for measuring usability for hard- and software products. It consists of 10 items and its results range from 0 to 100. Adjective rating scales were used for better interpretability [17].

The Attrakdiff 2 questionnaire [18] measures pragmatic and hedonic quality of a product. It consists of 4 subcategories: pragmatic quality, hedonic quality identity, hedonic quality stimulation, and attractiveness. The evaluation of these attributes is based on the ratings of 28 items, each of which is an adjective rating scale, ranging from −3 to 3.

The ISONORM 9241/10 questionnaire [19,20] assesses ergonomic principles for software dialogues according to ISO standard 9241 part 10. It consists of 7 categories: suitability for the task, self-descriptiveness, controllability, conformity with user expectations, error tolerance, suitability for individualization, and suitability for learning. Each category is evaluated through five 7-step items, ranging from −3 to 3.

#### Reproducibility Study

To evaluate test-retest reliability of our acquisition method (final app version on tablet PC) for symptom drawings, we planned to include 25 of our patients in the second study. The rational for this sample size was that previous studies had shown excellent test-retest reliability of pain extent with intraclass correlation coefficients (ICCs) between 0.92 and 0.97 [6]. Aiming at a 95% CI width of 0.1 with an alpha level of .05, we used formula 6 from Giraudeau and Mary [21] for our sample size estimation, which showed that 15 patients would be enough. As we planned to run additional reproducibility analyses for the number of clusters and the symptom pattern, we decided to include 25 patients. Thus, we asked 26 of our 51 patients to repeat their data entry after finishing the first one (1 patient had to be excluded because the image was not saved by the tablet PC). The second data entry was started 20 min after the first one, a period during which participants filled out other pain questionnaires of the pain outpatient department. When preparing their first drawing, patients were not aware that they would have to draw a second one. To avoid interference with clinical routines and bias by the consultation, only those patients who had waiting periods of more than 20 min were included for a second symptom drawing.

### Data Analysis

#### Usability Analysis

Mean values, SDs, and two-tailed *t* tests for all types of questionnaires for patients and doctors were calculated using Microsoft Excel (Microsoft Corporation, Redmont, WA).

#### Reproducibility Analysis

We used a script written in Python 2.7 (Python Software Foundation [22]) to transform image data originally saved as Portable Network Graphics into Neuroimaging Informatics Technology Initiative file format [23]. Tools from FMRIB Software Library [24] were used to extract image information, such as symptom extent (number of pixels), number of clusters, intersection, and union of symptom clusters, all of which were restricted to within the body outline. We calculated Jaccard index of symptom patterns as well as a two-way, mixed model ICC (ICC(3,1); according to Shrout and Fleiss classification [25]) for symptom extent (overall number of pixels) and number of symptom clusters for each of the 25 test-retest pairs using Microsoft Excel (Microsoft Corporation) and Real Statistics Resource Pack software (Release 5.4.1) [26]. Results were calculated independently for each body view. We then assessed the maximum and average values of all body views for each patient. Body views not used by the patients in both test and retest were excluded from the analysis. In case the patient drew multiple symptoms, these were merged for reproducibility analysis and the maximum VAS value was used for each pixel. Symptom extent was further plotted as a Bland-Altman plot (ie, mean difference of the drawings of each patient against the mean of both drawings) [27] using Microsoft Excel (Microsoft Corporation).

## Results

### Usability Evaluation

#### Patients’ Evaluation

A total of 52 questionnaires entered the final analysis for study 1 and 51 for study 2. In study 1, 87% (45/52) of the study patients were content with the body outline. In total, 8% (4/52) of the patients proposed changes in its size and 4% (2/52) requested gender-specific changes. Moreover, 75% (39/52) of the patients agreed with the available choice of descriptors, whereas 23% (12/52) asked for additional or different terms to describe their sensations. In addition, 71% (37/52) of the patients were contempt with the terms to describe the depth of their sensations, whereas 27% (14/52) were not. Several patients used the free text option to suggest adding a multiselect option for sensations and depth descriptors. Furthermore, free text entries demanded the possibility to rate multiple symptom intensities over the day or criticized the restriction of the drawings to within the borders of the body outline. All translated free text answers of patients’ usability questionnaire can be found in Multimedia Appendix 3. Finally, 23% (12/52) of all patients of study 1 stated that they had used the zooming option. However, only 6% (3/52) of all participating patients used the magnification buttons for this.

The part of the questionnaire comparing the app versions of studies 1 and 2 showed significant differences for 3 of the 8 Likert-type items, indicating improved usability of the final app (Table 3): (1) The difficulty of the drawing process decreased from 3.38 (SD 2.89) to 1.86 (SD 2.16; *P*=.003), (2) the ability to identify oneself with the given body outline increased from 7.54 (SD 2.59) to 8.73 (SD 1.71; *P*=.007), and (3) the difficulty to select a depth descriptor decreased from 4.71 (SD 3.18) to 3.27 (SD 2.77; *P*=.02).

In study 2, 4% (2/51) of the patients found the size of the body outline too small. One patient of 51 (2%) reported problems using the VAS while drawing and another one proposed to use a table to support the tablet during data collection. Finally, the difficulty of drawing the symptom pattern from different angles of the human body was rated as 1.78 (SD 2.16) on a Likert-type scale from 0 (“not difficult at all”) to 10 (“very difficult”).

**Table 3:** Usability assessment by patients comparing app versions from study 1 to study 2.

Likert-type questions	Study 1		Study 2		*P* value
	N	Mean (SD)	N	Mean (SD)	
How precisely does your drawing represent your actual sensations? (0=very imprecisely, 10=very precisely)	52	7.31 (2.33)	51	7.20 (3.05)	.84
How difficult was it to draw your sensations? (0=not difficult at all, 10=very difficult)	52	3.38 (2.89)	51	1.86 (2.16)	.003^a^
How well could you identify yourself with the body outline? (0=not at all, 10=very well)	52	7.54 (2.59)	51	8.73 (1.71)	.007^a^
How precisely do the chosen terms describe the nature of your sensations? (0=very imprecisely, 10=very precisely)	52	6.58 (2.54)	51	7.20 (2.47)	.21
How difficult was it to evaluate the depth of your sensations (ie, skin, muscle, etc)? (0=not difficult at all, 10=very difficult)	52	4.71 (3.18)	51	3.27 (2.77)	.02^a^
How precisely do you rate your drawing with the electronic pen in comparison with a pencil drawing? (0=very imprecise, 10=very precise)	52	7.52 (2.30)	51	7.10 (3.27)	.45
How much physical or mental stress was the drawing of your sensations? (0=no stress, 10=very much stress)	51	1.43 (2.18)	51	1.65 (2.21)	.62
How comprehensible were the drawing instructions (eg, drawing examples and written instructions) for you? (0=not comprehensible, 10=very comprehensible)	52	7.71 (2.41)	50	8.32 (2.07)	.18

^a^Statistically significant difference (*P*<.05).

**Table 4:** Usability assessment by doctors.

Questionnaire		Result (SD)
System Usability Scale (range 0 to 100)		75.63 (19.51)
**Attrakdiff 2 (Range −3 to 3)**		
	Pragmatic quality	1.07 (1.41)
	Hedonic quality: identity	1.14 (1.08)
	Hedonic quality: stimulation	1.25 (1.00)
	Attractiveness	1.14 (0.93)
**ISONORM 9241/10 (range −3 to 3)**		
	Suitability for the task	1.00 (1.62)
	Self-descriptiveness	0.95 (1.50)
	Controllability	−0.05 (1.43)
	Conformity with user expectations	1.25 (1.59)
	Error tolerance	0.65 (1.31)
	Suitability for individualization	−0.15 (1.79)
	Suitability for learning	1.80 (1.24)

#### Doctors’ Evaluation

The results of the doctors’ usability assessment are displayed in (Table 4). We collected data through the Web-based questionnaire from the 4 doctors participating in study 2. The mean score on the SUS was 75.63 (SD 19.51), indicating good usability. The subscales of the Attrakdiff questionnaire all received ratings between 1.07 and 1.25. Both the hedonic (self-centered) and pragmatic (action-oriented) quality of the tablet app had similar values. There was no indication of hedonic or pragmatic dominance in the final app.

Assessment using the ISONORM 9241/10 questionnaire showed good results in the categories “suitability for learning,” “suitability for the task,” “self-descriptiveness,” “conformity with user expectations,” and “error tolerance.” Only in the categories “suitability for individualization” and “controllability” the results were slightly below the average.

### Reproducibility Analysis

**Figure 2:**
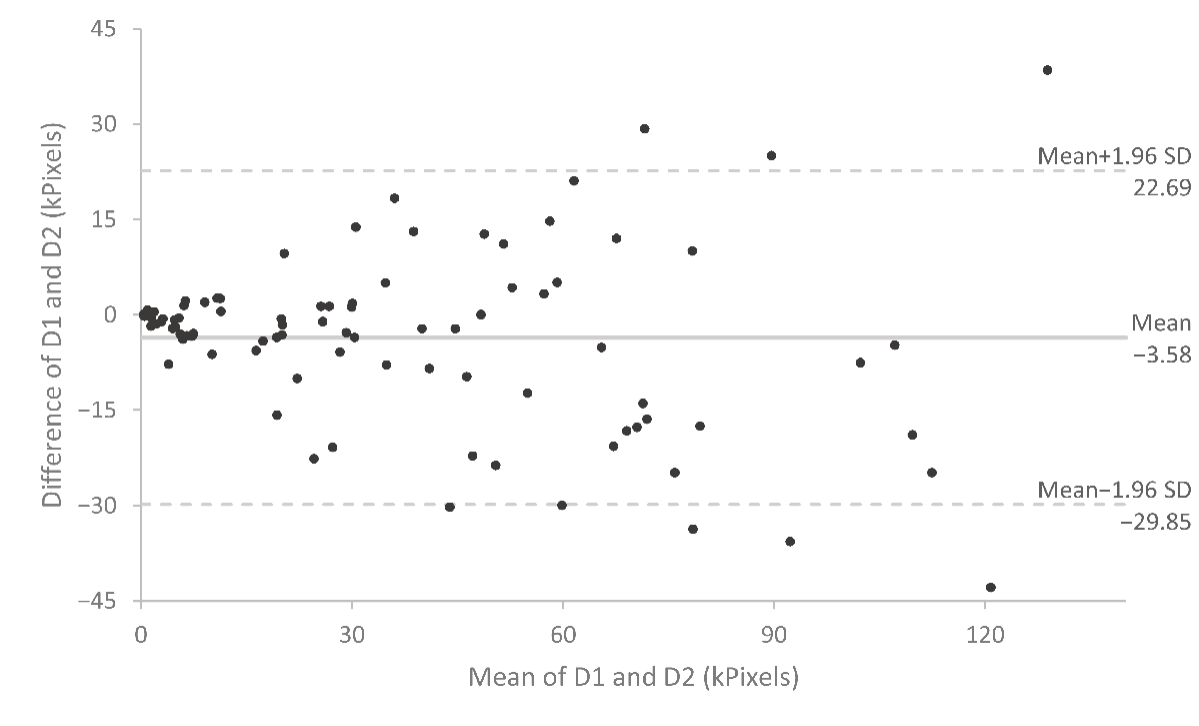
Bland-Altman plot of symptom extent. The central bold lines represent the mean difference. The dotted lines represent the 95% upper and lower limits of agreement. The mean symptom extent of the first and second symptom drawing (D1 and D2) is plotted against the difference in symptom extent between D1 and D2.

**Figure 3:**
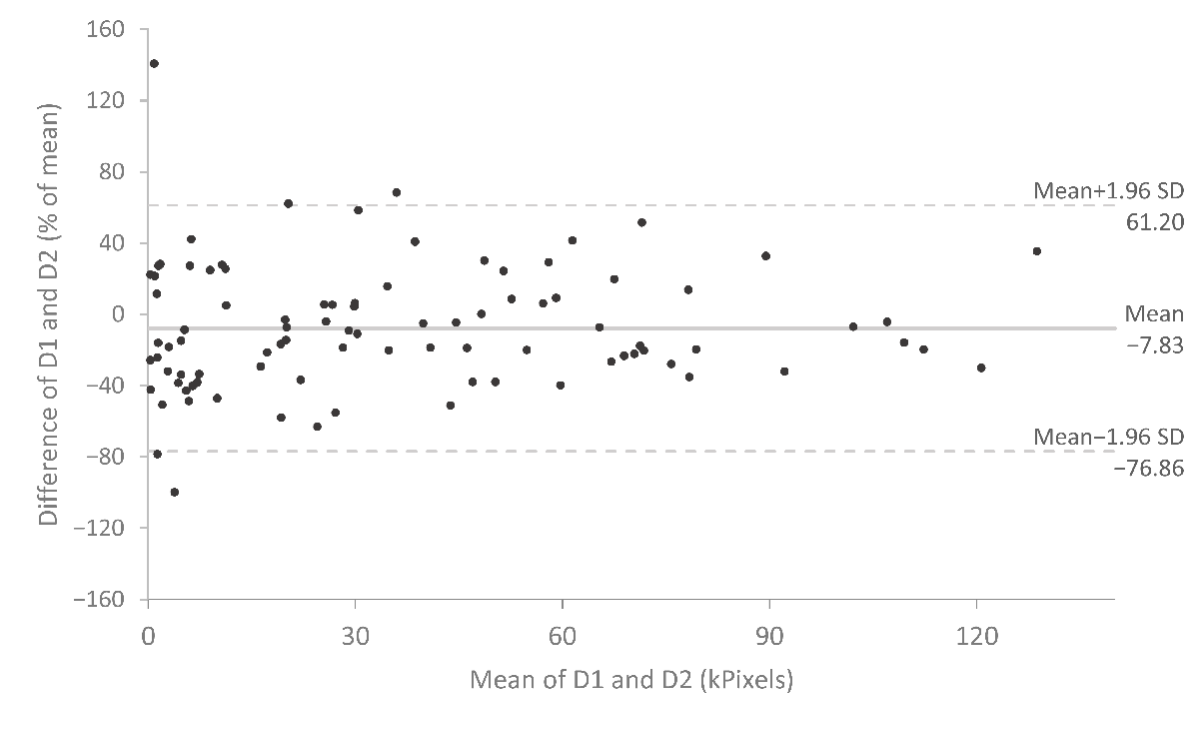
Bland-Altman plot of symptom extent. The central bold lines represent the mean difference. The dotted lines represent the 95% upper and lower limits of agreement. The mean symptom extent of first and second symptom drawing (D1 and D2) is plotted against the percentual difference (of the mean) between D1 and D1.

**Figure 4:**
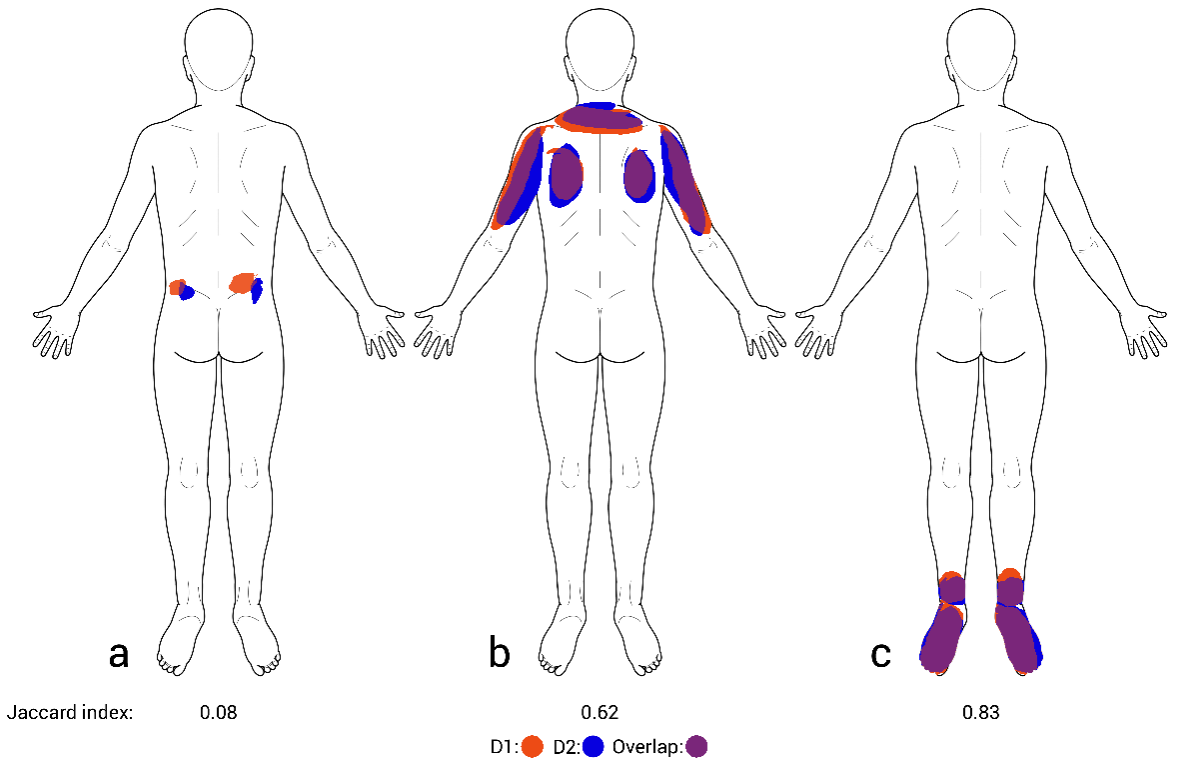
Test-retest reliability results and problems with the Jaccard index exemplified by symptom drawings of 3 different patients: (a) low, (b) average, (c) high reliability. The first drawing (D1) of each patient is colored in red and the second drawing (D2) in blue. Purple color indicates the overlap of the 2 drawings. Jaccard indices calculated from the consecutive drawings are reported below each drawing. From a clinical standpoint, D1 and D2 would still lead to the same clinical judgment.

Results of the test-retest analyses are shown in Table 5 and Figures 2-4. The Jaccard index was 0.47, indicating a mean overlap between test and retest symptom patterns of almost 50%. The ICC showed excellent reproducibility for symptom extent (ICC=0.92) and good reproducibility for Cluster count (ICC=0.70). Bland-Altman plots for symptom extent are shown in Figure 2 and Figure 3. There was no indication of a systematic difference between the measurements. Patients who drew larger areas also showed higher variability between test and retest drawings.

**Table 5:** Test-retest reliability.

Analysis			Result
Jaccard index of symptom pattern (SD)			0.47 (0.22)
**ICC^a^ of symptom extent (95% CI)**			
	Whole drawing (all body views)		0.92 (0.88-0.95)
	**Single views**		
		Front	0.93 (0.84-0.97)
		Back	0.90 (0.78-0.96)
		Left	0.94 (0.86-0.97)
		Right	0.92 (0.82-0.97)
**ICC number of symptom clusters (95% CI)**			
	Whole drawing (all body views)		0.70 (0.58-0.79)
	**Single views**		
		Front	0.66 (0.36-0.83)
		Back	0.56 (0.20-0.79)
		Left	0.75 (0.49-0.89)
		Right	0.87 (0.73-0.94)

^a^ICC: intraclass correlation coefficient.

## Discussion

We have developed a new tablet-based app (SymptomMapper) to collect electronic drawings of pain and related bodily symptoms. Following the suggested design guidelines by Jaatun et al [11], we limited written textual instruction, used a paper metaphor, and avoided pop-ups and other fast changes on the screen. Two versions of the app were tested for their usability and reproducibility in a sample of chronic pain patients and their treating doctors.

### Usability Evaluation

#### Patients’ Evaluation

Usability assessment comparing the pilot app from study 1 with the final app from study 2 showed 3 areas of significant improvement as rated by the patients: evaluation of symptom depth, identification with the body outline, and overall difficultness of the drawing process. Although we did not assess explicitly which changes of the app led to a particular improvement in usability, we will speculate in the following sections on the most likely causes and discuss them in the light of the relevant literature.

Regarding the improvement in depth evaluation, we must note that unless the pain is entirely superficial, a pain map has to display the complex three-dimensional geometry of the painful area onto a flat, two-dimensional surface [5]. It can be assumed that this is the main reason why depth assessment has never played a major role in pain drawings despite being used from the very beginning. In the famous McGill pain questionnaire, the letters E and I were used in the pain drawing part to distinguish between “external” and “internal” pain [3], whereas Margoles used the letter D to identify deep pain [28]. Jamison et al tested depth assessment within a three-dimensional assessment of pain drawings [29], and recently, Tucker et al have developed a visual rating instrument to assess the depth of experimental back pain by calculating the “percentage of depth to center” [30]. They could show that depth and lateral position may be the most critical descriptors to determine the source of acute lumbar muscular pain. Indeed, the differentiation between different layers is not only significant in regard to diagnostic purposes but also to different therapeutic approaches, such as in acupuncture, where needles are placed in different structures according to the underlying condition. Concerning evaluation of symptom depth, we made 2 related improvements, namely, splitting the depth category “skin” into “on the skin” and “beneath the skin,” and adding the possibility to choose multiple depth descriptors at once. The latter option was used by the majority of patients in study 2. In total, 26 out of 51 patients (51%) selected more than one depth category.

Concerning identification with the body outline and overall difficultness of the drawing process, we believe that the improvements seen here were largely due to 2 major modifications we made to the drawing module, namely, the introduction of gender-specific body outlines and the consecutive rather than joint presentation of the single body views. The change from a genderless to a gender-specific body outline had been requested by patients and may have improved patients’ ability to identify themselves with the body outline. Egsgaard et al have shown that gender aspects of body outlines can influence the quality of the drawing as well as the patients’ drawing experience [31]. In their study, 85% of the female study population preferred a female body chart.

On the other hand, the consecutive presentation of the body views constitutes a more guided approach compared with the joint presentation allowing patients to focus on one view at a time. This may also have improved identification with the body outline as compared with the pilot app. Finally, the size of the body outline was almost twice as large in the consecutive presentation approach as compared with joint presentation. Interestingly, however, the enlargement of the body outline did not have an effect on patients’ perceived exactness of the drawings.

#### Doctors’ Evaluation

At the end of study 2, the 4 participating doctors were asked to evaluate the tablet app through a Web-based usability survey consisting of the SUS, Attrakdiff 2, and ISONORM 9241/10 questionnaires. A score of 75.63 on the SUS indicates an overall good usability of the final app [17,32], which is in line with the results from the patients’ evaluation. With the Attrakdiff 2 questionnaire, we assessed pragmatic and hedonic qualities of the app, 2 dimensions that are independent from each other. Pragmatic quality perception evaluates the effectiveness of a product (task-related), whereas hedonic quality evaluates how the user-product interaction is stimulating the user and how the product is communicating the identity of the user (nontask-related) [33,34]. Attractiveness is the perceived property that is influenced by both pragmatic and hedonic qualities. However, although the pragmatic and hedonic qualities of a product are usually not altered by repeated use, its attractiveness can change, which Hassenzahl explains by a different weighting of these qualities based on the intention of the product usage [35]. We expected higher pragmatic than hedonic ratings, because the layout of the app was clearly task-oriented. However, the ratings of hedonic and pragmatic quality both showed comparable ratings around 1 on the scale from −3 to 3. According to these results, the final app as rated by doctors was neither a solitary “self-product” nor a pure “act-product” [34]. In general, the app received positive evaluations in all categories of the Attrakdiff 2.

The ISONORM 9241/10-questionnaire evaluates several aspects of software usability including suitability for the task, controllability, conformity with user expectations, and error tolerance. All categories were evaluated above average by our doctors, except for “suitability for individualization” and “controllability.” Both categories received slightly negative ratings, which may reflect the fact that doctors and patients used the same app in our study that only differed in the lists of available symptoms. As one of the design goals of this app was to increase homogeneity and validity of symptom drawings, we tried to reduce sources of between-subject variability that are due to unnecessary freedom in the drawing process. Therefore, the final app version used a rather rigid succession of data entry steps, for example, by presenting all body views in a defined order of by fixing the symptom descriptor(s) once the drawing starts. A possible way to improve this in future releases would be to add features that allow doctors to circumvent some of the restrictions and to enter data more freely.

### Reproducibility Analysis

After testing and improving the usability of our app, we used its final version to assess test-retest reliability of symptom drawings by a typical sample of chronic pain patients consulting a pain outpatient clinic. The analysis of pairs of consecutive drawings separated by 20 min and drawn by the same patients showed excellent reproducibility for symptom extent (ICC=0.92) and fair reproducibility for the exact symptom pattern (Jaccard index: 0.47). It is worth noting that we used 4 instead of the usual 2 views of the human. Although this complicates the drawing process, it allows for much more detailed assessment of lateral body regions. To our knowledge, this was the first study analyzing test-retest reliability for more than 2 body views.

Our results closely replicate a study by Barbero et al [6], who in 2 samples of pain patients (chronic low back pain and neck pain) reported an ICC of 0.92 and 0.97, respectively, for pain extent and a Jaccard index of 0.46 and 0.49, respectively, for pain location. We can also confirm their observation that the difference between the first and the second drawing increases with the total number of pixels drawn, whereas the percentual difference is constant. As Barbero et al note further, it is questionable if the Jaccard index is the optimal measure to assess the reliability of pain location as it demands very high precision in pain reporting. We completely agree with the authors here. Figure 4 shows the test-retest results of 3 of our study patients to illustrate this. There is no doubt that the first and second drawings of patients (b) and (c) with Jaccard indices of 0.62 (average) and 0.83 (high) would be considered identical from a clinical point of view. However, although a Jaccard index of 0.08 as exhibited by patient (a) indicates a very low test-retest reliability, his drawings still seem to convey the same clinical information. Thus, further investigations on symptom drawings and their association with clinical judgment are warranted. Furthermore, more useful measures of similarity that do not rely on exact overlap are needed.

### Limitations

As in every study, we must note some limitations. Our attempt to quantify symptom depth relied on verbal descriptors, which may have led to mistakes arising from different interpretations of expressions, such as “beneath the skin,” whose German translation may have been interpreted by some as meaning “in every tissue layer beneath the skin.” Descriptors should be carefully checked for all possible meanings they convey. Furthermore, there are alternative approaches to assess symptom depth, for example, the aforementioned visual rating of the “percentage of depth to center” by Tucker et al [30] or depth assessment based on three-dimensional pain drawings as used by Jamison et al [29].

Our test-retest results may have been biased by several uncontrolled factors. First, patients did not simply wait for 20 min between test and retest but used the time to fill out clinical pain questionnaires in preparation for their appointment with the doctors. The occupation with different aspects of their pain induced by the questionnaires may have influenced the second drawing, for example, by reminding patients of previously forgotten pain foci. Second, we cannot estimate the effect of learning. Roach et al showed that patients may complete pain drawings more reliably after they have been exposed to them several times [36]. In our study, patients only drew twice, and none of them underwent a training for using the app correctly, except for the drawing instructions given on the first screen. Finally, some patients reported during their second drawing that they had forgotten a symptom or complete body view in the first drawing. Despite the negative impact on test-retest reliability, we decided against excluding these patients, because their behavior probably reflects that of general pain patients.

### Conclusions

We developed an app for symptom drawing acquisition and assessed the usability of it in a sample of chronic pain patients and their treating doctors. We measured increases in usability of the improved app in terms of identification with the body outline, symptom depth evaluation, and difficultness of the drawing process according to patients’ evaluation (Aim 1). Furthermore, usability evaluation through treating doctors showed good overall usability and balanced hedonic and pragmatic values (Aim 2). Test-retest reliability of symptom drawings by chronic pain patients showed fair to excellent reproducibility for symptom pattern, symptom extent, and number of symptom clusters (Aim 3). Patients’ usability evaluation is an important factor when designing apps for mobile or eHealth apps and should not be neglected.

## Appendix

Multimedia Appendix 1 GRRAS checklist for reporting of studies of reliability and agreement.

Multimedia Appendix 2 Usability questionnaires (patients).

Multimedia Appendix 3 Free text answers of usability questionnaires (patients).

## Abbreviations

D1: first symptom drawing
D2: second symptom drawing
ICC: intraclass correlation coefficient
ISCED: International Standard Classification of Education
PC: personal computer
SUS: System Usability Scale
VAS: Visual analog scale
