# Supplementary material for: Designing a Tablet-Based Software App for Mapping Bodily Symptoms: Usability Evaluation and Reproducibility Analysis
Source: JMIR Mhealth Uhealth. 2018 May 30;6(5):e127. doi: 10.2196/mhealth.8409 (PMC6000481; doi:10.2196/mhealth.8409)
Supplement: Multimedia Appendix 1 [file mhealth_v6i5e127_app1.pdf]

Multimedia Appendix

This is a Multimedia Appendix to a full manuscript published in the J Med Internet Res. For full copyright and citation information see http://dx.doi.org/10.2196/mhealth.8409.

GRRAS checklist for reporting of studies of reliability and agreement

GRRAS checklist for reporting of studies of reliability and agreement

Section		check	Section in manuscript / Comment
Title and Abstract	1. Identify in title or abstract that interrater/intrarater reliability or agreement was investigated.	✓	Abstract
Introduction	2. Name and describe the diagnostic or measurement device of interest explicitly.	✓	Introduction
	3. Specify the subject population of interest.	✓	Introduction, Methods (Study design; Study participants)
	4. Specify the rater population of interest (if applicable).		Image analysis software, specified in Methods (Data analysis)
	5. Describe what is already known about reliability and agreement and provide a rationale for the study (if applicable).	✓	Introduction
Methods	6. Explain how the sample size was chosen. State the determined number of raters, subjects/objects, and replicate observations.	✓	Methods (Outcome measures - Reproducibility study)
	7. Describe the sampling method.	✓	Methods (Study design; Study participants - Pain patients; Outcome measures - Reproducibility study)
	8. Describe the measurement/rating process (e.g. time interval between repeated measurements, availability of clinical information, blinding).	✓	Methods (Outcome measures - Reproducibility study)
	9. State whether measurements/ratings were conducted independently.	✓	Methods (Outcome measures - Reproducibility study)
	10. Describe the statistical analysis.	✓	Methods (Data analysis - Reproducibility study)
	11. State the actual number of raters and subjects/objects which were included and the number of replicate observations which were conducted.	✓	Methods (Outcome measures - Reproducibility study)
Results	12. Describe the sample characteristics of raters and subjects (e.g. training, experience).	✓	Introduction, Methods (Study design; Study participants), Discussion (Limitations)
	13. Report estimates of reliability and agreement including measures of statistical uncertainty.	✓	Results (Reproducibility analysis; Table 5; Figure 2)
Discussion	14. Discuss the practical relevance of results.	✓	Discussion (Reproducibility analysis, Limitations)
Auxiliary material	15. Provide detailed results if possible (e.g. online).	✓	Results (Reproducibility analysis); Raw data can be requested from the authors.

This table is modified from Table 1 in Kottner J, Audigé L, Brorson S, Donner A, Gajeweski BJ, Hróbjartsson A, Roberts C, Shoukri M, Streiner DL. Guidelines for reporting reliability and agreement studies (GRRAS) were proposed. J Clin Epidemiol 2011;64(1):96-106. doi:10.1016/j.jclinepi.2010.03.002
