# Supplementary material for: Designing a Tablet-Based Software App for Mapping Bodily Symptoms: Usability Evaluation and Reproducibility Analysis
Source: JMIR Mhealth Uhealth. 2018 May 30;6(5):e127. doi: 10.2196/mhealth.8409 (PMC6000481; doi:10.2196/mhealth.8409)
Supplement: Multimedia Appendix 2 [file mhealth_v6i5e127_app2.pdf]

Multimedia Appendix

This is a Multimedia Appendix to a full manuscript published in the J Med Internet Res. For full copyright and citation information see http://dx.doi.org/10.2196/mhealth.8409.

Usability questionnaires (Patients)

Study 1
p. 2-4 (FSA-01)
Study 2
p. 5-6 (FSA-02)

Usability Questionnaire:

Subject-ID: ________________________

1. How precisely does your drawing represent your actual sensations?										
Very imprecisely										Very precisely
○ 0	○ 1	○ 2	○ 3	○ 4	○ 5	○ 6	○ 7	○ 8	○ 9	○ 10

2. How difficult was it to draw your sensations?										
Not difficult at all										Very difficult
○ 0	○ 1	○ 2	○ 3	○ 4	○ 5	○ 6	○ 7	○ 8	○ 9	○ 10

3. How well could you identify yourself with the body outline?										
Not at all										Very well
○ 0	○ 1	○ 2	○ 3	○ 4	○ 5	○ 6	○ 7	○ 8	○ 9	○ 10

4. What would you change in the given body outline?						
○ nothing	○ thicker	○ thinner	○ taller	○ shorter	○ more masculine	○ more feminine
Other:						

5. How precisely do the chosen terms describe the nature of your sensations?										
Very imprecisely										Very precisely
○ 0	○ 1	○ 2	○ 3	○ 4	○ 5	○ 6	○ 7	○ 8	○ 9	○ 10

6. Would you have wished for more or different terms to describe your sensations?	
○ yes	○ no
If yes, which?	

7. How difficult was it to evaluate the depth of your sensations (i.e. skin, muscle, etc.)?										
Not difficult at all										Very difficult
○	○	○	○	○	○	○	○	○	○	○
0	1	2	3	4	5	6	7	8	9	10

8. Would you have wished for more or different terms to describe the depth of your sensations?	
○ yes	○ no
If yes, which?	

9. Did you use one of the magnification tools of the application?		
○ yes	○ no	
If yes, which?		
○ Magnifier	○ Two-finger-zoom	○ both

10. How precisely do you rate your drawing with the electronic pen in comparison with a pencil drawing?										
Very imprecise										Very precise
○	○	○	○	○	○	○	○	○	○	○
0	1	2	3	4	5	6	7	8	9	10

11. How often do you use comparable electronic devices (e.g. computers, tablet computers, or smartphones)?					
○	○	○	○	○	○
daily	3-4 times /week	1-2 times /week	1-2 times /month	Almost never	Never

12. How much physical or mental stress was the drawing of your sensations?										
No stress										Very much stress
○	○	○	○	○	○	○	○	○	○	○
0	1	2	3	4	5	6	7	8	9	10

13. How comprehensible were the drawing instructions (e.g. drawing examples and written instructions) for you?										
Not comprehensible										Very comprehensible
○	○	○	○	○	○	○	○	○	○	○
0	1	2	3	4	5	6	7	8	9	10

14. Did you have any problems when drawing your sensations?

15. What would you improve?

Usability Questionnaire:

Subject-ID: ________________________

1. How precisely does your drawing represent your actual sensations?										
Very imprecisely										Very precisely
○	○	○	○	○	○	○	○	○	○	○
0	1	2	3	4	5	6	7	8	9	10

2. How difficult was it to draw your sensations?										
Not difficult at all										Very difficult
○	○	○	○	○	○	○	○	○	○	○
0	1	2	3	4	5	6	7	8	9	10

3. How well could you identify yourself with the body outline?										
Not at all										Very well
○	○	○	○	○	○	○	○	○	○	○
0	1	2	3	4	5	6	7	8	9	10

4. How difficult was it to draw from the different perspectives?										
Not difficult at all										Very difficult
○	○	○	○	○	○	○	○	○	○	○
0	1	2	3	4	5	6	7	8	9	10

5. How precisely do the chosen terms describe the nature of your sensations?										
Very imprecisely										Very precisely
○	○	○	○	○	○	○	○	○	○	○
0	1	2	3	4	5	6	7	8	9	10

6. How difficult was it to evaluate the depth of your sensations (i.e. skin, muscle, etc.)?										
Not difficult at all										Very difficult
○	○	○	○	○	○	○	○	○	○	○
0	1	2	3	4	5	6	7	8	9	10

7. How precisely do you rate your drawing with the electronic pen in comparison with a pencil drawing?										
Very imprecise										Very precise
○	○	○	○	○	○	○	○	○	○	○
0	1	2	3	4	5	6	7	8	9	10

8. How often do you use comparable electronic devices (e.g. computers, tablet computers, or smartphones)?					
◯ daily	◯ 3-4 times /week	◯ 1-2 times /week	◯ 1-2 times /month	◯ Almost never	◯ Never

9. How much <u>physical or mental stress</u> was the drawing of your sensations?										
No stress										Very much stress
◯ 0	◯ 1	◯ 2	◯ 3	◯ 4	◯ 5	◯ 6	◯ 7	◯ 8	◯ 9	◯ 10

10. How comprehensible were the <u>drawing instructions</u> (e.g. drawing examples and written instructions) for you?										
Not comprehensible										Very comprehensible
◯ 0	◯ 1	◯ 2	◯ 3	◯ 4	◯ 5	◯ 6	◯ 7	◯ 8	◯ 9	◯ 10

11. Did you have any <u>problems</u> when drawing your sensations?

12. What would you improve?
