# Supplementary material for: Designing a Tablet-Based Software App for Mapping Bodily Symptoms: Usability Evaluation and Reproducibility Analysis
Source: JMIR Mhealth Uhealth. 2018 May 30;6(5):e127. doi: 10.2196/mhealth.8409 (PMC6000481; doi:10.2196/mhealth.8409)
Supplement: Multimedia Appendix 3 [file mhealth_v6i5e127_app3.pdf]

Multimedia Appendix

This is a Multimedia Appendix to a full manuscript published in the J Med Internet Res. For full copyright and citation information see http://dx.doi.org/10.2196/mhealth.8409.

Free text answers of usability questionnaires (Patients)

Study 1
p. 2-3
Study 2
p. 4

Study 1 - Free text answers

Question 4: What would you change in the given body outline?

- The display of the jaw/oral cavity

Question 6: Would you have wished for further or different terms to describe the nature of your sensations? If yes, which?

- Multiple answer options; variable pain intensity
- Multiple terms
- Continuous persistent pain
- Inner trembling
- Multiple terms + Illustrating the relationships of pain cycles
- tugging
- Selection of multiple descriptors.
- Detailed, textual pain description (e.g. cranial segments, cranial region, spinal column of the cranial region up to cervical spine)
- Easier multiple selection e.g. temporal cramps
- boring; paralyzing
- Multiple answer options
- My tingling is radiating to the upper arm, but in the hand it is all the time. That was not representable.

Question 8: Would you have wished for further or different terms to describe the depth of your sensations? If yes, which?

- More direct
- Neuropathic pain
- Vessels
- Detailed body parts/zones
- Beginning + end of depth
- Where the pain radiates
- Other color marks
- Direction of radiation
- Multiple selection
- Organ, (here penis root)

Question 14: Did you have any problems when drawing your sensations?

- Toothache + jaw pain were not representable
- none
- For generalized sensations => Chills (minor symptoms)
- none
- Order: First body section, then pain area would be better!
- With instructions and support almost no problems
- The markings disappeared outside of the manikin. Thus, one has to draw the inner outline of the manikin with great care
- Describing pain with adjectives
- none
- No.
- Size
- To define depth with only one term

Question 15: What would you improve?

- That several terms can be entered for a pain sensation
- Question, when pain is stronger or weaker (time of day)
- none
- Multiple terms the type of sensation
- See notes (e.g. develop 4th page for the notes (e.g. pain depth))
- nothing at all
- No.
- Magnification
- Motor symptoms

Study 2 - Free text answers

Question 11: Did you have any <u>problems</u> when drawing your sensations?

- none
- None
- Drawing migraine pain
- None
- None
- No problems
- none
- not at all
- Small
- None
- none
- none
- The pain-affected regions are changing - sometimes the left arm, next day => right leg! Not one region constantly.
- Figure was a bit too small

Question 12: What would you improve?

- Nothing
- It is okay like this
- nothing
- nothing right now
- everything ok.
- Multiple colors
- nothing!
- Everything ok. --> Maybe provide a clipboard as support or a desk.
- The provided descriptors for the pain were not entirely matching my pain characteristic (i.e. incomplete)
- Separate basic needs like sleep or eating, since when someone in artificially fed, they have massive problems in this area but not with sleep, occupation, etc.
- Pain perception is not equally strong in all marked regions -> e.g shoulders/neck 7 -> knee 3!
- everything ok
- Zooming in on single body regions
